# NIR Laser-Responsive PNIPAM and Gold Nanorod Composites for the Engineering of Thermally Reactive Drug Delivery Nanomedicine

**DOI:** 10.3390/pharmaceutics12030204

**Published:** 2020-02-27

**Authors:** Yejin Kwon, Yonghyun Choi, Jaehee Jang, Semi Yoon, Jonghoon Choi

**Affiliations:** School of Integrative Engineering, Chung-Ang University, Seoul 06974, Korea; angang1027@gmail.com (Y.K.); dydgus5057@gmail.com (Y.C.); jjaeh95@gmail.com (J.J.); semi103306@gmail.com (S.Y.)

**Keywords:** poly(*N*-isopropylacrylamide), gold nanorod, doxorubicin, drug delivery, NIR laser

## Abstract

When ingesting a drug on its own or injecting it directly into tissue, its concentration increases immediately within the body, which often exacerbates the side effects and increases its toxicity. To solve this problem, we synthesized the thermally reactive polymer poly(*N*-isopropylacrylamide) (PNIPAM) using reversible addition–fragmentation chain transfer (RAFT) polymerization and prepared nanocarriers by binding PNIPAM to gold nanorods (GRs), with the anticancer agent doxorubicin (DOX) used as a model drug. PNIPAM changes from hydrophilic to hydrophobic at temperatures above its lower critical solution temperature, which represents a coil-to-globule volume phase transition. Because GRs absorb near-infrared (NIR) laser light and emit energy, PNIPAM aggregation occurs when the synthesized PNIPAM/GR are subjected to an NIR laser, and the temperature of the GRs rises. Using this principle, DOX was combined with the PNIPAM/GR complex, and the resulting anticancer effects with and without laser treatment were observed in Hela and MDA-MB-231 cells. In our proposed complex, the GR binding rate of PNIPAM reached 20% and the DOX binding rate reached 15%. The release profile of the drug following laser irradiation was determined using a drug release test and confocal microscopy imaging. It was subsequently confirmed that the release of the drug is higher at higher temperatures, especially with laser treatment. The proposed combination of temperature-reactive polymers and gold nanostructures shows promise for future research into controlled drug release.

## 1. Introduction

Surgery, radiation therapy, and chemotherapy are the most common treatments for cancer worldwide, but chemotherapy in particular has various side effects, including strong cytotoxicity caused by the drugs attacking normal cells [1,2,3]. Therefore, many researchers have looked to develop drug formulations that control the rate of release [4,5,6,7] in order to maintain constant concentrations of the drug in the blood or target organ [8]. To control the release rate, nanocarriers that release the drug when exposed to either internal or external stimuli have been proposed. A number of these nanocarriers are synthesized using stimuli-reactive polymers [9], especially poly(*N*-isopropylacrylamide) (PNIPAM), which is a well-known temperature-reactive polymer [10,11].

Temperature-reactive polymers all have a lower critical solution temperature (LCST), and that of PNIPAM ranges from 30 to 45 °C [12,13]. PNIPAM undergoes a phase change at the LCST. In an aqueous solution, PNIPAM is hydrophilic at temperatures below the LCST, but aggregation occurs at temperatures above the LCST when PNIPAM becomes hydrophobic [14,15]. The change in the LCST of PNIPAM depends on the ions present in the solution. In general, the biological and chemical effects of ions in aqueous solutions depend on the Hofmeister series (kosmotropes, such as sodium carbonate, sodium sulfate, phosphate, and chloride ions, and chaotropes, such as nitrate, perchlorate, and thiocyanate) [16]. The change in the LCST of PNIPAM when certain ions are present is due to the increase in the surface tension between the hydrophobic moieties (i.e., the isopropyl group and hydrocarbon backbone) of the PNIPAM and adjacent hydration water. It also occurs as a result of the weakening of the hydrogen bonds between the hydration water and the amide groups of the PNIPAM via the polarization effect [17].

**Figure 1:**
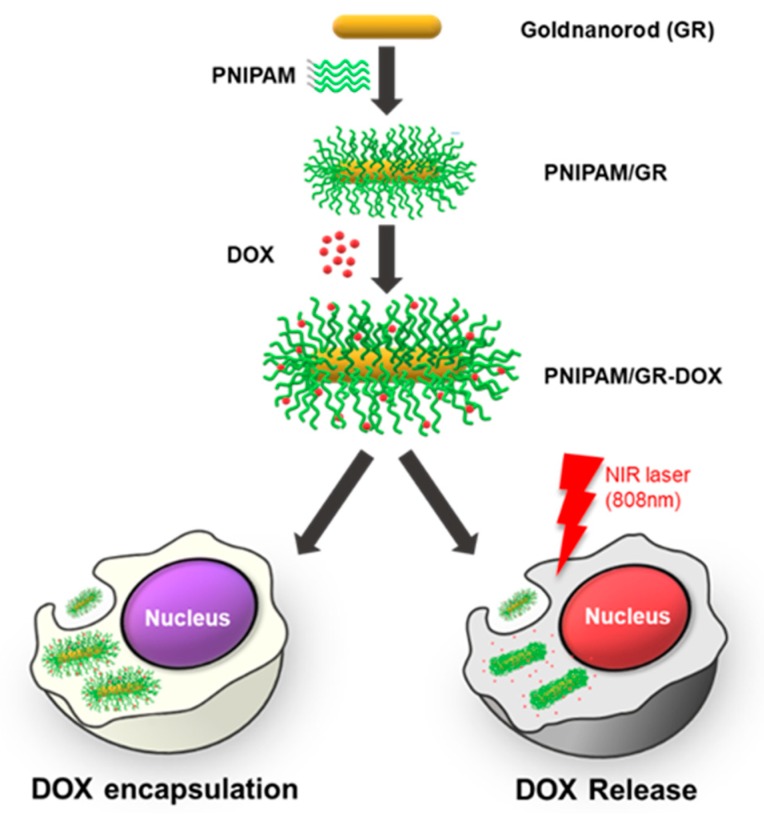
Schematic illustration of the poly(*N*-isopropylacrylamide–gold-nanorod–doxorubicin (PNIPAM/GR-DOX) complex used to selectively release an anticancer drug into cancer cells when exposed to near-infrared (NIR) laser irradiation.

Gold nanorods (GRs) have demonstrated great potential for use in drug delivery and photothermal therapy (PTT) [18,19] Spherical gold nanoparticles (AuNPs) have a light absorption range of 400–600 nm; however, very little of the light in this range is able to pass through the skin, thus their PTT effect is very low [20]. In contrast, GRs have the unique optical properties of strong light absorption and heat emission in the visible–near-infrared (NIR) range (750–1000 nm) depending on their size and aspect ratio [21,22,23]. The GRs are designed to absorb light at 808 nm, a wavelength that has high skin penetration [24]. However, GRs are highly cytotoxic due to the hexadecyltrimethyl ammonium bromide (CTAB) present on their surface, which is used to maintain dispersion [25,26]. Low concentrations of CTAB in cells do not affect cell growth, but high concentrations cause cell membrane damage, leading to cell death [27]. To overcome this cytotoxicity, other studies have used polymers [28,29] and phospholipid molecules [30] to coat the surface of GRs. In the present study, stability is maintained by coating the surface of the GRs with PNIPAM to reduce the toxicity. Figure 1 illustrates the synthesis process for the final complex (PNIPAM/GR-DOX) and the release of the anticancer drug doxorubicin (DOX) from the compound under NIR laser irradiation.

In order to bind PNIPAM to the GRs, the trithiocarbonate groups of the PNIPAM were converted to thiol groups. After the synthesis of the PNIPAM/GR complex, DOX was noncovalently bound to the PNIPAM/GR. PNIPAM becomes hydrophobic when the GRs are irradiated with an NIR laser because the GRs absorb light and the temperature of the surrounding microenvironment subsequently rises above the LCST of the polymer. The hydrophobic–hydrophobic interaction leads to aggregation and releases the DOX. In this study, we tested the efficacy of the proposed temperature-reactive drug carrier using an NIR laser as a trigger to release the drug molecules.

## 2. Materials and Methods

### 2.1. Materials

*N*-isopropylacrylamide (NIPAM), 2-(dodecylthiocarbonothioylthio)-2-methyl propionic acid (DDMAT), 2,2′-azobis(2-methylpropionitrile) (AIBN), ethanolamine, tris(2-carboxyethyl) phosphine hydrochloride (TCEP·HCl), gold(III) chloride trihydrate (HAuCl_4_·3H_2_O), CTAB, silver nitrate (AgNO_3_), l-ascorbic acid (AA), doxorubicin hydrochloride, Trypan blue solution, Triton X-100, paraformaldehyde, glassware, RC dialysis membrane tubing (6–8 kD molecular weight cut-off, Spectra/Por), and 1,4-dioxane were all purchased from Sigma-Aldrich (St. Louis, MO, USA). Tetrahydrofuran (THF), *n*-hexane, methanol, and toluene were purchased from Daejung Chemicals (Seoul, Korea). Purified water used to synthesize PNIPAM/GR, and PNIPAM/GR-DOX was purified using a Millipore Milli-Q system (18.2 MΩcm resistance). Hela and MDA-MB-231 cells were obtained from Korea Cell Link Bank (Seoul, Korea). NIPAM monomers were recrystallized using a mixture of hexane and toluene (7:3, v/v), and AIBN was recrystallized using methanol, followed by PNIPAM synthesis. Cell culture dishes, glass-bottomed dishes, and 96-well cell culture plates were purchased from SPL Life Sciences (Gyeonggi-do, Korea). Dulbecco’s modified Eagle medium (DMEM, high glucose) and Dulbecco’s phosphate-buffered saline (DPBS) were purchased from Pan Biotech (Aidenbach, Germany). Fetal bovine serum (FBS) and Antibiotic–Antimycotic (100×) were obtained from Gibco, ThermoFisher (Waltham, MA, USA). A Cell Counting Kit-8 (CCK-8) was purchased from Dogenbio (Seoul, Korea).

### 2.2. Synthesis of PNIPAM-SH

**Figure 2:**
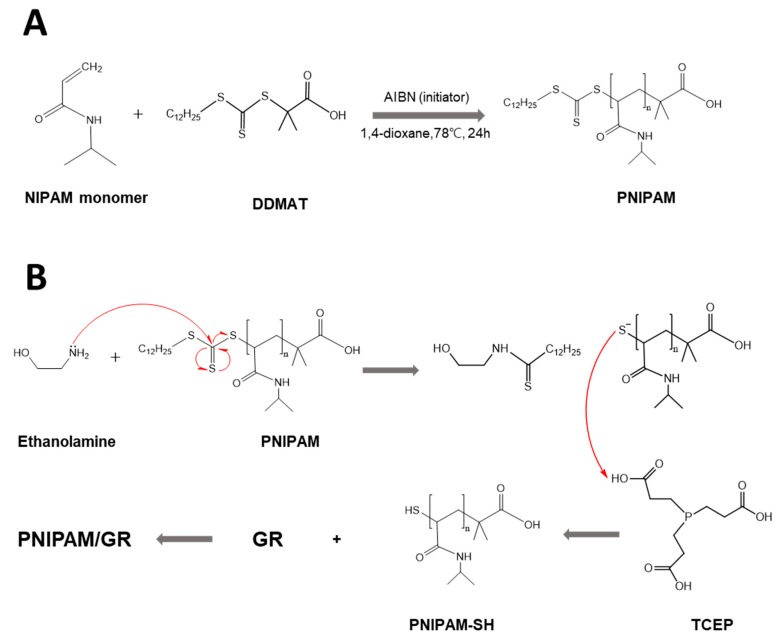
Chemical reaction protocol for the synthesis of PNIPAM and PNIPAM-SH. (**A**) Synthesis of PNIPAM using reversible addition–fragmentation chain transfer (RAFT) polymerization. (**B**) Aminolysis for the preparation of thiol-terminated PNIPAM.

First, we used reversible addition–fragmentation chain transfer (RAFT) polymerization to synthesize PNIPAM. RAFT polymerization allows for the accurate control of molecular weight and polydispersity. It can also synthesize block copolymers with complex structures that are otherwise difficult to generate [31]. Various studies have reported the synthesis of PNIPAM using RAFT polymerization [32,33], and we modified their protocols slightly for the present study. NIPAM monomers (17 g, 1.5 mmol) purified using a mixture of toluene and hexane were dissolved in 11 mL of 1,4-dioxane, followed by the addition of DDMAT (30.3 mg, 0.075 mmol) as a RAFT agent and AIBN (3 mg, 0.002 mmol) as an initiator (Figure 2A). Oxygen was removed from the solution for 30 min using Ar gas, and the reaction allowed to proceed for 24 h at 78 °C. A two-neck flask containing the synthesized PNIPAM solution, which corresponds to the termination of RAFT polymerization, was placed in ice and 10 mL of *n*-hexane was stored in ice in a 40–60 mL beaker to make it cold. Recrystallization of NIPAM was performed by dissolving 5 g NIPAM monomer in a 100 mL solution with n-hexane and toluene in a ratio of 7:3. The filter paper was used to obtain pure NIPAM. The recrystallization of AIBN was accomplished by dissolving 5 g of AIBN in 200 mL methanol and in the same way as NIPAM. The solidified polymer was dissolved again in 1,4-dioxane and then recrystallized in cold hexane three times to remove impurities. Dialysis was then carried out for three days at 4 °C, followed by lyophilization to obtain a powder. The average molecular weight (Mn) of the synthesized PNIPAM was 1.5 × 10^4^, and the polydispersity index (PDI) was 1.32. As shown in Figure 2B, the synthesized PNIPAM contained a trithiocarbonate group that was converted to a thiol group via aminolysis. In this process, the trithiocarbonate group is removed using nucleophiles (e.g., amines, thiols, and hydroxides) and ionic reducing agents (e.g., boron hydrides), during which side reactions occur to form disulfide bonds [34]. To prevent this, the disulfide on the surface is typically reduced using a reducing agent such as sodium bisulfite (Na_2_S_2_O_4_) or phosphine-reducing agent (TCEP) [35]. Synthesized thiol-terminated polymers are commonly conjugated with AuNPs [36] or biopolymers [37].

The synthesized PNIPAM (200 mg, 10 μmol) was dissolved in THF, and the oxygen was then removed using Ar gas. Ethanolamine (17 μL, 300 μmol) and TCEP (0.9 mg, 3.3 μmol) were subsequently added to the solution. Additional Ar gas purging was carried out for 15 min, and the solution was left to sit for 24 h at 27 °C to allow the reaction to occur. Impurities were removed in the same manner as for the PNIPAM. The M_n_ of the synthesized PNIPAM-SH compound was 1.4 × 10^4^, and the PDI was 1.36.

### 2.3. Synthesis of PNIPAM/GR

AuNPs are often combined with polymers to increase their stability [38]. There are typically two approaches for the binding of polymers to AuNPs: “grafting to”, a method of binding already-synthesized polymers to AuNPs, and “grafting from”, in which the polymer is grown on the surface of the AuNPs using a surface-confined living radical polymerization process. In the present study, we grafted PNIPAM-SH onto GRs using the “grafting-to” sulfur–gold interaction.

The GRs were synthesized following a protocol reported elsewhere [39]. AgNO_3_, HAuCl_4_, and ascorbic acid were added to CTAB to produce Au seeds. After reacting at 27 °C for 24 h, the supernatant was removed using centrifugation at 13,000× *g* for 15 min. DI water was then added, and centrifugation was repeated three times (washing and isolation). The obtained GRs were stored in the refrigerator at 4 °C.

In order to bind the PNIPAM-SH to the GRs, 1 mL of GRs in solution and 10 mg of PNIPAM-SH were mixed, and then DI water was added so that the total volume of the solution was 5 mL. This solution was reacted in the dark for 42 h at 260 RPM at 4 °C. Centrifugation was then performed twice for a total of 15 min at 11,000× *g* to remove unbound PNIPAM. The resulting complex was then dispersed in water for a total volume of 1 mL.

### 2.4. Preparation of PNIPAM/GR-DOX

DOX binds to PNIPAM/GR nanoparticles through electrostatic interactions. Before synthesizing the PNIPAM/GR-DOX complex, experiments were carried out using UV–vis spectra to determine whether the synthesized GRs had a wavelength near 808 nm. For this, 200 μL of DOX (2 mg/mL) was added to 1 mL of the synthesized PNIPAM/GR complex, and a solution with a total volume of 5 mL prepared. The solution was reacted using a shaker in the dark for 30 h at 4 °C. After the reaction, centrifugation was conducted twice for 15 min each at 11,000× *g* to remove any unbound DOX. The resulting complex was dispersed in water for a total volume of 1 mL.

### 2.5. Characterization of PNIPAM/GR-DOX

#### 2.5.1. ^1^H-NMR Spectrometry (600 MHz)

To characterize the synthesized PNIPAM-SH complex, a VNS NMR spectrometer (Varian, Palo Alto, CA, USA) was used. About 30 mg of the sample were dissolved in 0.5 mL of CDCl_3_, an NMR analysis solvent, and stored in an NMR tube for measurement. Subsequent analysis was conducted using the MestReNova program (Mestrelab Research, Compostela, Spain).

#### 2.5.2. Gel Permeation Chromatography

In order to confirm the molecular weight of the synthesized PNIPAM and PNIPAM-SH complexes, gel permeation chromatography (GPC) with a KF-803 chromatograph (Shodex) was used. A mixture of chloroform and 1% triethylamine (TEA) was used as a solvent. The flow rate was 1 mL/min, the temperature was 35 °C, and the injection volume was 50 µL.

#### 2.5.3. UV–Vis Spectroscopy

A total of three different UV–vis spectroscopes were used to analyze various characteristics of the synthesized complexes. First, a V-770 spectrophotometer (JASCO, Tokyo, Japan) was used to analyze the UV–vis spectra of the PNIPAM and PNIPAM-SH complexes. PNIPAM and PNIPAM-SH were dissolved in DI water at 0.5% (w/w) and measured within the 200–600 nm wavelength range. Second, to confirm the LCST of PNIPAM and PNIPAM-SH, absorbance was measured by increasing the temperature by 1 °C from 20 to 60 °C at a wavelength of 350 nm using a Cary 100 Bio spectrophotometer (Varian). To determine the LCST in three solvents, PNIPAM and PNIPAM-SH were measured by dissolving them at 0.05% (w/w) in DI water, 1× PBS, and DMEM. Finally, the absorbance for wavelengths from 300 to 1000 nm was measured using a Biomate 3S spectrophotometer (ThermoFisher, Waltham, MA, USA) to determine the maximum absorption wavelength of the GRs, PNIPAM/GR, and PNIPAM/GR-DOX.

#### 2.5.4. Inductively Coupled Plasma Mass Spectrometry

In order to confirm the concentration of the synthesized GRs, analysis was performed using a NexION 350D inductively coupled plasma mass spectrometer (ICP-MS; Perkin-Elmer, Waltham, MA, USA). The synthesized GRs were measured by diluting them 100 times.

#### 2.5.5. Field Emission Transmission Electron Microscopy

In order to confirm the binding of PNIPAM to the GRs, GRs and PNIPAM/GR were observed using a JEM-F200 field emission transmission electron microscope (FE-TEM; JEOL Ltd., Tokyo, Japan). The GR and PNIPAM/GR solutions were diluted to a concentration of about 50 μg/mL, with several 10-μL aliquots then dropped onto a copper grid. The PNIPAM/GR complex was stained with uranyl acetate to allow the PNIPAM to be easily observed. In order to confirm the aggregation of PNIPAM/GR following NIR laser treatment, the grid containing PNIPAM/GR was subjected to NIR laser light and subsequently observed using the FE-TEM.

#### 2.5.6. Assessment of DOX Loading Efficiency

Aliquots of DOX solution (1 mL) were diluted to 400, 200, 100, 50, 25, 12.5, and 6.25 μg/mL and measured as a standard for UV–vis absorbance analysis. Absorbance was measured at 490 nm to obtain a standard calibration curve with a coefficient of determination (R^2^) of 0.9998.

To assess the efficacy of the synthesized PNIPAM/GR as a drug carrier, the loading efficiency of PNIPAM/GR-DOX was measured. After DOX had been attached to the PNIPAM/GR, unbound DOX was removed and the supernatant stored separately during the isolation process. Absorbance was then measured using a multi-plate reader (Synergy H1, BioTek, Winooski, VT, USA). The amount of bound DOX was calculated by comparing the absorbance with the standard DOX curve, and the drug-loading efficiency was calculated using the following Equation (1):

(1) $$\mathrm{Doxorubicin}\;\mathrm{loading}\;\mathrm{efficacy}\;(\%)=\frac{\mathrm{Initial}\;\mathrm{DOX}\;\mathrm{Conc}.\;-\;\mathrm{unbound}\;\mathrm{DOX}\;\mathrm{Conc}.}{\mathrm{Initial}\;\mathrm{DOX}\;\mathrm{Conc}.}\times 100$$

#### 2.5.7. Fourier-Transform Infrared Spectroscopy

To confirm that the synthesized complexes were strongly bound, the absorbance peaks for PNIPAM, PNIPAM-SH, PNIPAM/GR, PNIPAM/GR-DOX, and free DOX were measured using a Fourier-transform infrared spectrometer (FTIR; Alpha II, Bruker, Billerica, MA, USA).

#### 2.5.8. Zeta Potential Analysis

A Zetasizer Nano Zs (Malvern, Malvern, UK) was used to measure the charge on the surface of the nanoparticles. GRs coated with CTAB, PNIPAM/GR, and PNIPAM/GR-DOX were diluted in DI water and the surface charge measured.

#### 2.5.9. Temperature Change of GRs and PNIPAM/GR in DMEM under Laser Irradiation

In order to measure the changes in temperature under laser irradiation, GR and PNIPAM/GR solutions were first diluted to concentrations of 100, 50, 10, 5, or 1 μg/mL in a 96-well plate. The wells were irradiated using an infrared laser pointer (808 nm 1500 mW 1.5 W Infrared Laser Pointer, BeamQ, San Francisco, USA) for 10, 20, 30, 60, 120, 180, and 240 s and the change in the temperature of the solution recorded.

#### 2.5.10. Drug Release Profile of PNIPAM/GR-DOX

In order to investigate the drug release profile of PNIPAM/GR-DOX according to the temperature of the microenvironment, the release of DOX was measured over time at 25 and 40 °C. PNIPAM/GR-DOX (1 mL) was placed in a dialysis bag and transferred to 5 mL of tertiary distilled water. The amount of DOX released was then measured using fluorescence analysis (excitation: 480 nm; emission: 590 nm).

#### 2.5.11. Cell Viability Assays

Before demonstrating the anticancer effect of PNIPAM/GR-DOX, the cytotoxicity of the nanoparticles was assessed using Hela and MDA-MB-231 cells. The Hela and MDA-MB-231 cells were cultured in an incubator at 37 °C under 5% CO_2_ in high-glucose DMEM containing 10% FBS, 1% penicillin, and streptomycin. Each cell type was seeded at a concentration of 5 × 10^3^ cells/well in a 96-well plate and incubated at 37 °C under 5% CO_2_ for 24 h. PNIPAM and PNIPAM-SH were independently diluted in DMEM to concentrations of 500, 250, 125 and 62.5 μg/mL, while the GRs and PNIPAM/GR were diluted to 25, 12.5, 6.25, and 3.125 μg/mL in DMEM based on the GR concentration. Cytotoxicity was measured using CCK-8 assays after 24 and 48 h in an incubator at 34 °C under 5% CO_2_.

The Hela and MDA-MB-231 cells were used to assess the anticancer effect of PNIPAM/GR-DOX. The cells were cultured as described above at a concentration of 5 × 10^3^ cells/well in a 96-well plate and incubated at 37 °C under 5% CO_2_ for 24 h. PNIPAM/GR-DOX and free DOX were then added at concentrations of 2.0, 1.0, 0.5, and 0.25 μg/mL based on the concentration of DOX. Each sample was incubated at 34 °C under 5% CO_2_ for 2 h and then illuminated with an 808-nm laser for 30, 50, 80, or 150 s depending on the concentration of DOX. The cytotoxicity was measured after incubation at 34 °C under 5% CO_2_ concentration for 24 and 48 h.

For PNIPAM/GR treatment, the cells were cultured in 96-well plates and treated with various PNIPAM/GR concentrations. The Hela cells were then irradiated with the laser for 15, 30, 50 and 80 s, while the MDA-MB-231 cells were irradiated for 30, 50, 80 and 150 s.

#### 2.5.12. Confocal Laser Scanning Microscopy

Confocal imaging experiments were conducted to confirm the cell uptake of the synthesized PNIPAM/GR-DOX and its anticancer effects. Hela cells were incubated in high-glucose DMEM containing 10% FBS, 1% penicillin, and streptomycin at 37 °C under 5% CO_2_. The cells were then seeded at a concentration of 1 × 10^4^ cells/dish in a confocal dish and incubated at 37 °C under 5% CO_2_ for 24 h. PNIPAM/GR-DOX and free DOX were added to the cells at 1 μg per 200 μL based on the DOX concentration and incubated at 34 °C under 5% CO_2_ for 2 h. They were then illuminated with an 808-nm laser for 90 s and stored in an incubator for 1 h. After removing the samples from the medium and washing them twice with DPBS, 2 mL of 4% paraformaldehyde was added. The samples were stored at 4 °C for 1 h, after which the paraformaldehyde was removed. The samples were washed three times with DI water and 1 mL of 1% Triton X-100 was added. They were stored for 30 min at 25 °C and washed four times with DI water. Mounting solution with DAPI solution was then added to the samples and refrigerated. An LSM710 confocal microscope (Carl Zeiss, Oberkochen, Germany) was used to measure the fluorescence of DAPI and DOX in the samples.

#### 2.5.13. Statistical Analysis

Two-tailed and unpaired *t*-tests were conducted in GraphPad Prism 7.0 for Windows (GraphPad Software, Inc., La Jolla, CA, USA) [40]. Non-significant values have been represented as ns, while *, **, ***, **** indicate *p*-values < 0.0332, 0.0021, 0.0002 and 0.0001, respectively.

## 3. Results and Discussion

We used aminolysis to transform the synthesized PNIPAM into PNIPAM-SH, and various analytical methods were used to confirm that the trithiocarbonate groups of PNIPAM had been completely converted into thiol groups.

**Figure 3:**
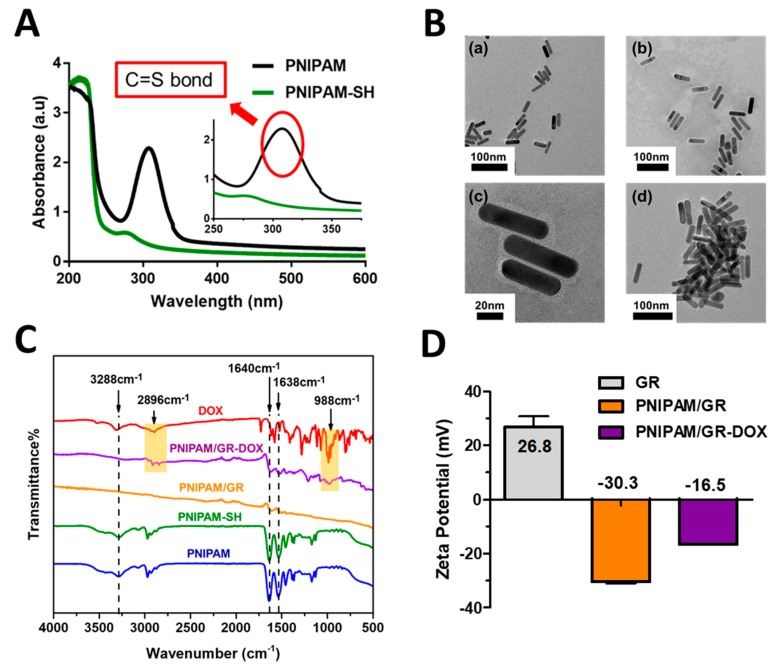
Analytical confirmation of PNIPAM, PNIPAM-SH, PNIPAM/GR, PNIPAM/GR-DOX synthesis. (**A**) UV–vis absorption spectra of PNIPAM and PNIPAM-SH in DI water. (**B**) FE-TEM images of (a) GRs, (b) and (c) PNIPAM/GR, and (d) PNIPAM/GR aggregations after laser treatment. Scale bars indicate 100 μm in (a), (b), (d) and 50 μm in (c). (**C**) FT-IR spectra of PNIPAM, PNIPAM-SH, PNIPAM/GR, PNIPAM/GR-DOX, and free DOX (wavenumber 500–4000 cm^−1^. The yellow shaded area illustrates DOX binding. (**D**) Surface zeta potential of the synthesized GR-CTAB, PNIPAM/GR, and PNIPAM/GR-DOX dispersed in DI water.

**Figure A1:**
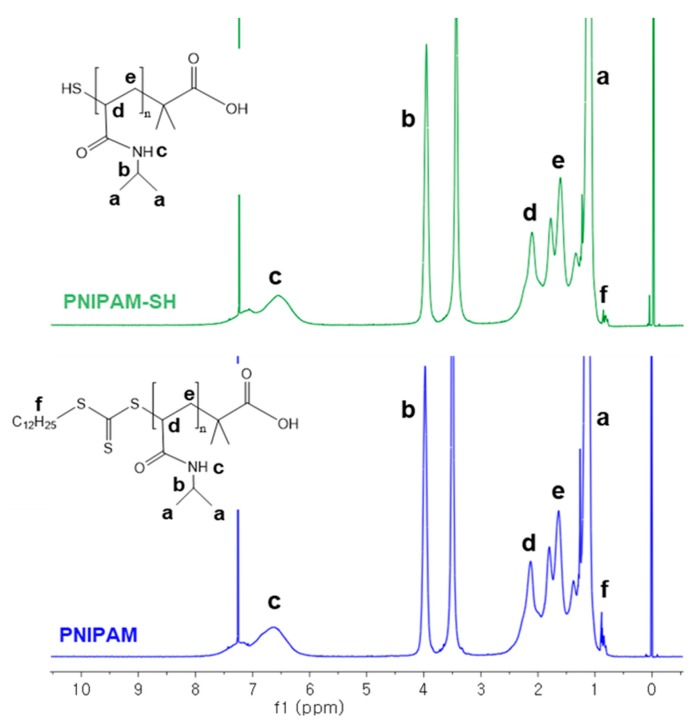
Comparison of the ^1^H-NMR (600 MHz) spectra for PNIPAM (lower graph, blue) and PNIPAM-SH (upper graph, green) in CDCl_4_.

In Figure 3A, it can clearly be observed that the UV–vis peak at around 310 nm corresponding to the trithiocarbonate group of PNIPAM was not present for PNIPAM-SH after aminolysis [33]. The structure of the synthesized polymer was also confirmed by ^1^H-NMR spectra (Figure A1). When comparing the spectra for PNIPAM and PNIPAM-SH, it can be seen that the peak is lower for PNIPAM-SH at 0.84–0.87 ppm due to the loss of –CH3 protons from the trithiocarbonate group via aminolysis [41,42].

**Figure A2:**
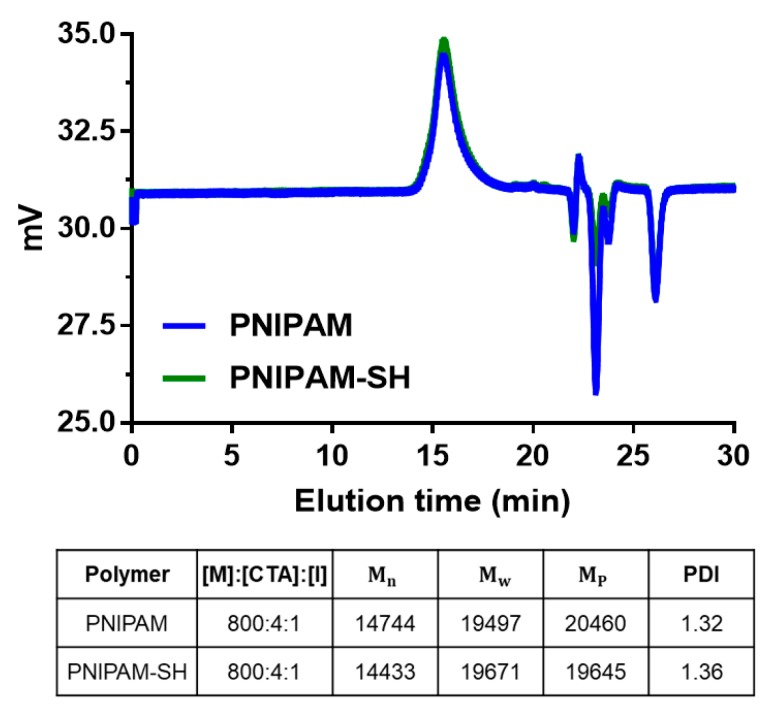
GPC analysis of PNIPAM and PNIPAM-SH using chloroform + TEA 1% as an eluent at a flow rate of 1.0 mL/min.

As described above, the removal of the trithiocarbonate groups from PNIPAM using ethanolamine leads to the formation of disulfide bonds, thus increasing the molecular weight. We measured the molecular weight of PNIPAM and PNIPAM-SH using GPC to demonstrate that the side reaction leading to the formation of disulfide bonds did not occur (Figure A2). The GPC results show that the M_n_ of PNIPAM-SH was slightly lower than that of PNIPAM after aminolysis. This means that no disulfide bonds were formed, that the trithiocarbonate groups were completely removed, and that PNIPAM-SH was successfully synthesized [34].

FE-TEM was employed to determine the size and shape of the synthesized GRs and PNIPAM/GRs (Figure 3B). The synthesized GRs had a long axis of 53.2 ± 2.1 nm and a short axis of 14.2 ± 0.8 nm, with a consistent shape. In the image of GRs coated with PNIPAM-SH presented in Figure 3B (upper right), a light gray film can be observed, meaning that a thin layer of PNIPAM-SH had bound to the GRs, while the enlarged image in Figure 3B (lower left) shows this film more clearly. It can be seen that the layer of PNIPAM-SH on the GRs was not uniform, with a thickness varying from 1 to 10 nm. Figure 3B (lower right) presents PANIPM/GR aggregations arising from the increase in temperature due to 808-nm laser radiation. Therefore, through FE-TEM, we demonstrated the change in PNIPAM/GR based on the shape of the GRs, the binding of PNIPAM-SH, and laser irradiation.

**Figure A3:**
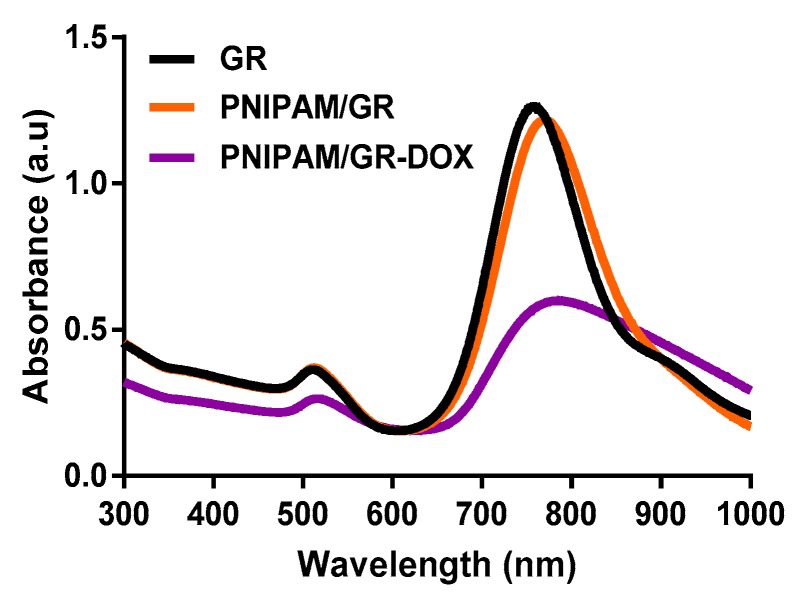
UV–vis spectra of GRs, PNIPAM/GR, and PNIPAM/GR-DOX.

Three methods were used to determine whether DOX had successfully been bound electrostatically to PNIPAM/GR. It was found that the binding rate of DOX was as high as 17%. The UV–vis spectra presented in Figure A3 show that the synthesized GRs had a surface plasmon resonance wavelength of 759 nm, while the PNIPAM/GR-DOX had a wavelength of 786 nm, representing a redshift of 27 nm. Thus, the peak for PNIPAM/GR-DOX was close to the 808 nm wavelength, which indicates that an 808-nm laser would be suitable for the excitation of the complex. Fourier-transform infrared (FT-IR) spectra confirmed the surface characteristics of the nanoparticles at all stages up to the synthesis of PNIPAM/GR-DOX as the final product (Figure 3C). The most representative peaks for PNIPAM were ascribed to N–H stretching from the secondary amines in the amine groups (3288 cm^−1^), the N–H bending peak (1638 cm^−1^), and the peak for C=O (1640 cm^−1^). PNIPAM-SH, PNIPAM/GR, and PNIPAM/GR-DOX all exhibited peaks near those of PNIPAM [43], while the C–H stretching peak (2896 cm^−1^) and the C–O stretching peak (988 cm^−1^) from the free DOX can be identified in PNIPAM/GR-DOX.

The zeta potential was also measured to determine the surface charge of the synthesized nanoparticles (Figure 3D). The GRs had a positive charge of 26.8 mV due to the presence of CTAB. When the PNIPAM-SH was coated onto the GR surface, the stability of the GR particles was maintained even when the CTAB was removed during the isolation process. Figure A3 shows that there is almost no peak difference between PNIPA-GR after 30 h of synthesis with GR. Therefore, gold particles do not aggregate and stability of particles is maintained. The zeta potential was −30.3 mV due to the PNIPAM on the GR surface, increasing to −16.5 mV with the addition of DOX due to the positively charged primary amine [44]. These results indicate that PNIPAM and DOX bound successfully to the GR surface.

**Figure 4:**
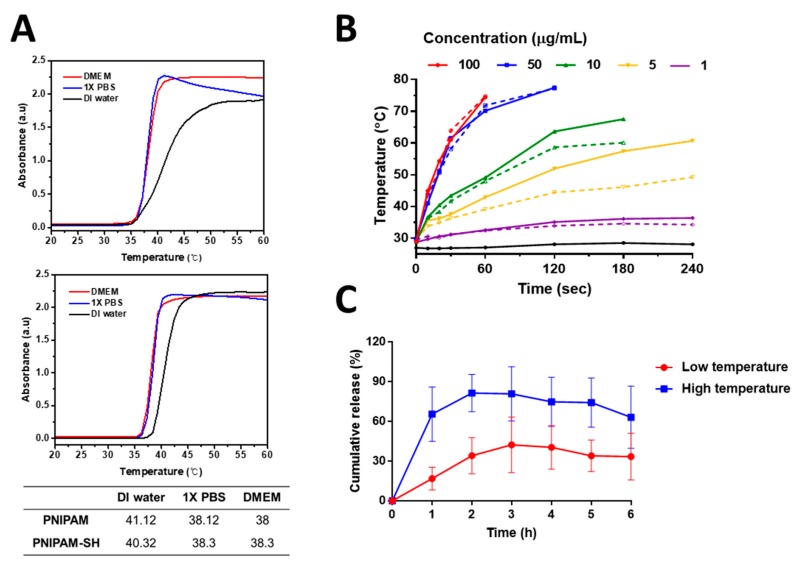
Thermo-characterization of PNIPAM, GRs, and PNIPAM/GR. (**A**) The lower critical solution temperature (LCST) of PNIPAM (upper graph) and PNIPAM-SH (lower graph) for various solutions measured using UV–vis absorption spectra. (**B**) Temperature changes for various particle concentrations and NIR irradiation times in DMEM (solid line: GRs; dotted line: PNIPAM/GR; black line: DMEM). (**C**) In vitro drug release profile for PNIPAM/GR-DOX and free DOX at different temperatures.

The LCST of PNIPAM is known to vary depending on the ions present. We investigated this by dissolving PNIPAM and PNIPAM-SH in various solvents. In Figure 4A, it can be seen that the LCST of PNIPAM and PNIPAM-SH in DMEM and PBS, which have a relatively high ion concentration, is lower than that of PNIPAM and PNIPAM-SH in DI water. GRs absorb light and convert it into heat [45], so we used an 808-nm laser to irradiate synthesized GRs and PNIPAM/GR and compare the change in temperature. Figure 4B shows that there was no significant difference in the change in temperature between GR and PNIPAM/GR. The laser irradiation time required for the temperature to rise above 40 °C with the GRs was about 10 s at the highest GR concentration (100 μg/mL). However, at the lowest concentration (1 μg/mL), laser irradiation for 300 s did not increase the temperature above 40 °C for either GRs or PNIPAM/GR. DMEM without GRs exhibited a temperature rise of about 1.2 °C after irradiation with the 808-nm laser for 240 s. Thus, we confirmed the heat release capability of the synthesized GRs.

We next investigated the DOX release behavior of PNIPAM/GR-DOX to determine whether it had the potential to be a suitable temperature-responsive drug carrier. The DOX release was assessed at a low temperature (25 °C) and a high temperature (above 40 °C) corresponding to the LCST of PNIPAM (Figure 4C). One hour after the start of the experiment, approximately 65% of the DOX had been released at the high temperature, compared to 17% at the low temperature. Above 40 °C, the maximum release was 80% in 2 h while, at 25 °C, the maximum was 42% in 3 h. These results show that the release efficiency of the drug was almost twice as high in the high-temperature environment, which means that PNIPAM/GR-DOX is thermally responsive and thus the release rate of the drug can be controlled by temperature.

**Figure A4:**
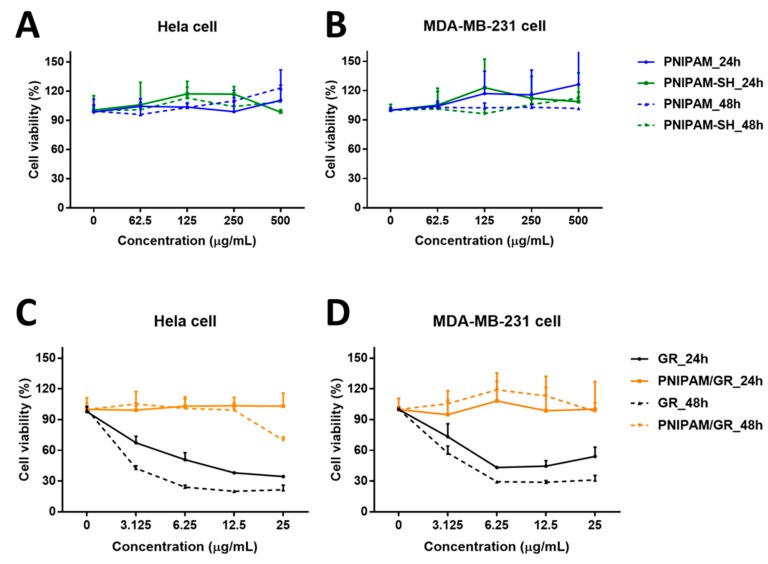
Cell viability following PNIPAM, PNIPAM-SH, GR, and PNIPAM/GR treatment. Cytotoxicity of PNIPAM and PNIPAM-SH in (**A**) Hela and (**B**) MDA-MB-231 cells. Cytotoxicity of GRs and PNIPAM/GR in (**C**) Hela and (**D**) MDA-MB-231 cells. Only the GRs exhibited significant toxicity.

We also evaluated the cytotoxicity of PNIPAM, PNIPAM-SH, GRs, and PNIPAM/GR after 24 and 48 h in Hela and MDA-MB-231 cells using CCK-8 assays. PNIPAM and PNIPAM-SH were not toxic after either 24 or 48 h at high concentrations (500 μg/mL) in either cell line (Figure A4A,B). This means that PNIPAM on its own demonstrates potential as a drug delivery agent. Figure A4C,D showed that the presence of GRs with CTAB led to a cell survival rate of 67.34% after 24 h for the Hela cells at the lowest concentration (3.125 μg/mL), decreasing to 42.4% after 48 h. In MDA-MB-231 cells, cell viability was 73.2% after 24 h and 57.4% after 48 h, thus demonstrating similar cytotoxicity to Hela cells. However, at a concentration 6.25 μg/mL, there was a sharp decrease in cell viability in both cell lines (50.8% for Hela and 43.2% for MDA-MB-231 cell survival after 24 h and 24.1% and 29.4% after 48 h, respectively). We coated PNIPAM onto the GR surface to reduce the CTAB toxicity. When exposed to PNIPAM/GR, the survival rate for Hela and MDA-MB-231 cells was 100% after 24 h at the highest concentration (25 μg/mL). After 48 h, the MDA-MB-231 cells still had a 100% cell viability, but the Hela cells had a 70% decrease in cell viability, down to 30% survival rate. However, the Hela cell survival rate was 100% after 48 h at concentrations below 12.5 μg/mL. This means that the cytotoxicity of CTAB was somewhat ameliorated during the isolation process when PNIPAM bound to the GRs, indicating that our synthesized PNIPAM/GR complex can be used for therapeutic purposes with minimal cytotoxicity.

**Figure 5:**
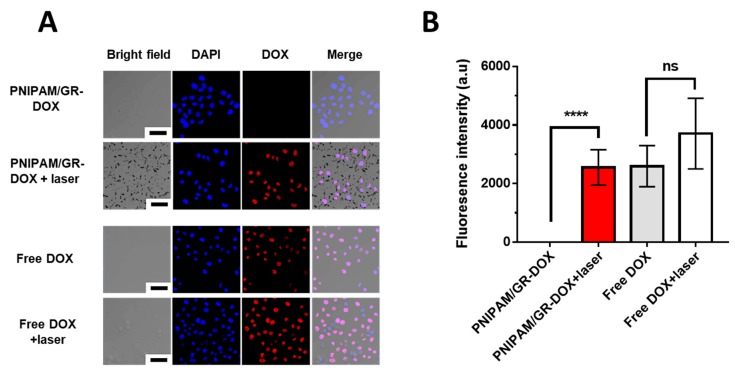
Confocal images of the Hela cell line treated with various nanoparticles with and without laser irradiation (808 nm, 1.5 W, 90 s). The nuclei are stained with DAPI (blue). Scale bar: 50 μm. (**A**) Confirmation of the cellular uptake of PNIPAM/GR-DOX and DOX at a DOX concentration of 0.5 μg/mL and of GR aggregation when PNIPAM/GR-DOX is irradiated with the laser. (**B**) DOX fluorescence intensity inside the cellular nucleus for various compounds. Non-significant values have been represented as ns, while **** indicates *p*-values < 0.0001.

We treated the Hela cells with either PNIPAM/GR-DOX or free DOX at a DOX concentration of 0.5 μg/mL to determine their effect on the cells in the presence or absence of laser irradiation using confocal laser scanning microscopy. Figure 5A shows that, for free DOX, the drug is taken into the nucleus within 2 h of the treatment. There was no significant difference in the fluorescence of DOX in the nucleus with or without NIR laser irradiation. However, for PNIPAM/GR-DOX, the DOX fluorescence inside the nucleus is notably stronger under laser irradiation. In addition, laser treatment led to the formation of PNIPAM/GR-DOX aggregations that were visible to the naked eye.

The levels of DOX inside the nucleus were then assessed based on the intensity of DOX fluorescence in the nucleus (Figure 5B). When treated with free DOX, there was no difference in fluorescence intensity with or without NIR laser treatment. When the cells were treated with PNIPAM/GR-DOX, there was almost no fluorescence in the nucleus with no laser treatment. With laser treatment, however, significantly higher fluorescence intensity was recorded in the nucleus. Thus, the thermal reactivity of PNIPAM/GR in the presence of laser irradiation was confirmed, leading to the controlled release of DOX and indicating that the proposed complex is effective in delivering drugs into the nucleus.

To confirm the anticancer effect of DOX released through the laser treatment of PNIPAM/GR-DOX, we examined cell viability using CCK-8 assays with Hela and MDA-MB-231 cells. The cells were treated with PNIPAM/GR, PNIPAM/GR-DOX, and free DOX at four different concentrations of DOX, and cell viability was assessed after 24 and 48 h. In order to induce the aggregation of PNIPAM, the temperature of the solution was raised to 40 °C by varying the irradiation time of the laser (808 nm, 1.5 W) depending on the DOX concentration.

**Figure 6:**
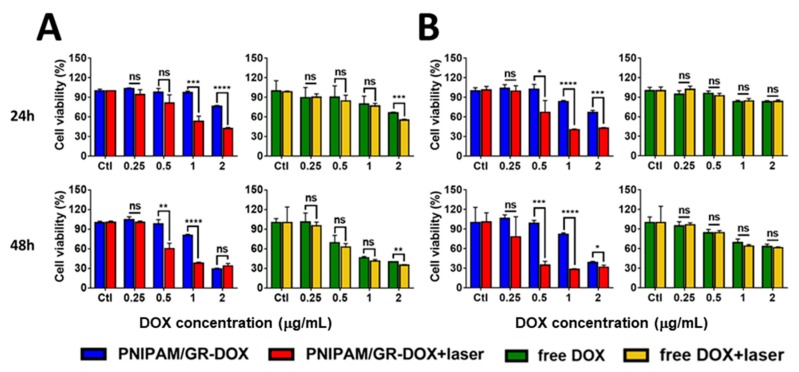
Confirmation of the anticancer effect after the chemo- and photo-thermal treatment of (**A**) Hela and (**B**) MDA-MB-231 cells for 24 and 48 h. To increase the temperature of the solution to 40 °C, the NIR laser (808 nm, 1.5 W) irradiation time differed for each DOX concentration (2 μg/mL: 30 s; 1 μg/mL: 50 s; 0.5 μg/mL: 80 s; 0.25 μg/mL: 150 s). The data represents the mean ± SD; *: *p* < 0.0332; **: *p* < 0.0021; ***: *p* < 0.0002; ****: *p* < 0.0001.

As shown in Figure 6A, cell viability was 97.2% after 24 h in Hela cells treated with PNIPAM/GR-DOX but no laser treatment at a DOX concentration of 1 μg/mL, while laser irradiation reduced cell survival to 53.1%. In contrast, treatment with free DOX led to a survival rate of 80% without laser treatment and 76.7% with laser treatment. The cell survival rate after 48 h was 80.4% with laser-free PNIPAM/GR-DOX treatment, but this decreased to 37.8% with the application of laser irradiation. As with the results after 24 h, there was little difference after 48 h in the cell survival rate when free DOX was used with and without laser treatment (41.0% and 46.1%. respectively).

As presented in Figure 6B, MDA-MD-231 cancer cells treated with PNIPAM/GR-DOX exhibited the greatest difference in cell viability after 24 h before and after laser treatment at a DOX concentration of 1 μg/mL (83.4% without and 40.3% with laser irradiation). Treatment with free DOX led to a cell viability of 83.3% without laser treatment and 84.2% with laser treatment, which was not statistically different. After 48 h, a DOX concentration of 0.5 μg/mL produced the greatest difference in cell viability before and after laser irradiation. With PNIPAM/GR-DOX treatment but no laser irradiation, the cell survival rate was 98.7%; the use of the laser reduced this to 64.0%. Again, there was no significant difference in the cell viability when free DOX was used with and without laser irradiation (84.6% and 84.2%, respectively).

**Figure A5:**
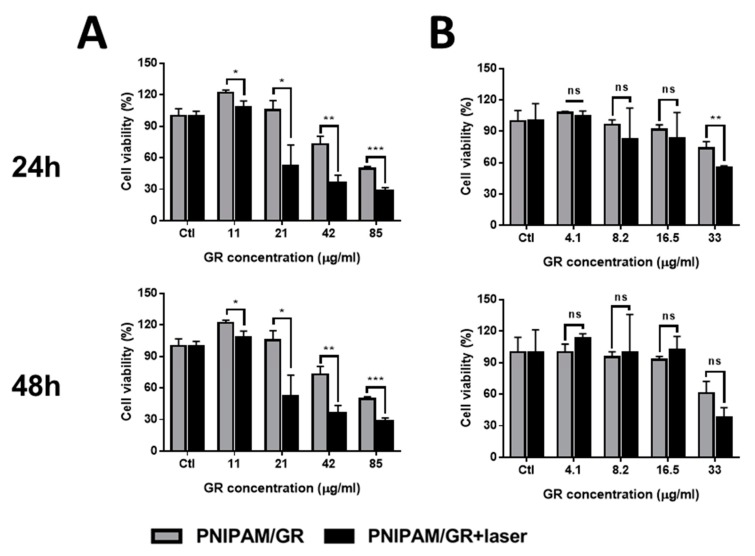
Cytotoxicity of PNIPAM/GR with NIR laser irradiation in (**A**) Hela and (**B**) MDA-MB-231 cells. Non-significant values have been represented as ns, while *, **, ***, **** indicate *p*-values < 0.0332, 0.0021, 0.0002 and 0.0001, respectively.

In summary, we demonstrated that the anticancer effect was higher with the application of laser irradiation to both Hela and MDA-MB-231 cells treated with PNIPAM/GR-DOX. The results of the experiments used to confirm the cell survival rate during the laser irradiation of PNIPAM/GR are summarized in Figure A5.

## 4. Summary

We synthesized a drug carrier by combining PNIPAM with AuNPs. PNIPAM is well-known as a temperature-reactive polymer, and we confirmed that the synthesized PNIPAM had hydrophilic properties at temperatures below 38 °C based on the LCST in DMEM. GRs were synthesized to maximize the light absorption from an 808-nm NIR laser, which offers the most effective skin penetration. We modified the end group of PNIPAM with a thiol to allow it to bind to the GRs. DOX, an anticancer agent, was subsequently bound to the PNIPAM/GR complex using electrostatic interactions. The successful formation of PNIPAM-SH was confirmed using ^1^H-NMR (600 MHz), UV–vis spectra and GPC, the successful synthesis of PNIPAM/GR was confirmed using TEM, and the binding of DOX to the PNIPAM/GR complex was confirmed using UV–vis spectra, FT-IR spectra, and the surface zeta potential. Following the successful fabrication of these complexes, Hela and MDA-MD-231 cells were directly exposed to PNIPAM, PNIPAM-SH, GRs, and PNIPAM/GR to assess their cytotoxicity. It was found that all of these except for the GRs were non-toxic. We then irradiated PNIPAM/GR-DOX using the 808-nm NIR laser to induce the aggregation of PNIPAM-SH due to the rise in temperature and to initiate the release of DOX. Confocal microscopy confirmed the release of DOX and the uptake of PNIPAM/GR-DOX into Hela cells following laser irradiation. CCK-8 assays also confirmed the anticancer effect of PNIPAM/GR-DOX and free DOX in both cancer cell lines. Treatment with free DOX exhibited no difference in cell viability with or without laser irradiation, whereas cell survival was up to 64% lower with PNIPAM/GR-DOX treatment when laser irradiation was employed. The proposed PNIPAM/GR-DOX complex was thus proven to effectively release DOX following irradiation with an 808-nm NIR laser and offers significant potential as a drug-delivery system. The advantage of this work over the previous work is that the chain reaction is designed to increase the hydrophobicity of PNIPAM in response to the increase in ambient temperature by GR absorbing light through the specific 808 nm wavelength. Structural contraction with increasing hydrophobicity was designed to release the DOX that was incorporated at this time. By controlling the rate of drug release through the laser, selective cancer cell death and reduced toxicity to normal cells can be expected.
